# Rapid and robust bioanalytical assays are critical for SARS-CoV-2 therapeutic and vaccine development and beyond

**DOI:** 10.4155/bio-2020-0116

**Published:** 2020-05-26

**Authors:** Sumit Kar, Rafiqul Islam

**Affiliations:** ^1^Bioanalytical Sciences, Celerion Inc. 621 Rose St, Lincoln, NE 68502, USA

**Keywords:** COVID-19, neutralizing antibodies, serology, viral load

Effective therapies and prophylactic vaccines for the severe acute respiratory syndrome coronavirus 2 (SARS-CoV-2) that is causing the coronavirus disease 2019 (COVID-19) pandemic are urgently needed. The scientific community is rapidly testing antivirals that target the ability of SARS-CoV-2 to infect and replicate including protease inhibitors, polymerase inhibitors and antimalarials [1]. Anti-inflammatory drugs are being tested to prevent lung injury from the virus. Antibodies or convalescent plasma antibodies can bind to unique epitopes and have different neutralizing mechanisms of action. Finally, prophylactic vaccines are being tested to produce an immune response against SARS-CoV-2.

Irrespective of the type of therapy, a suite of bioanalytical assays is required to characterize efficacy and safety. Even repurposed therapies need testing with new assays specific for SARS-CoV-2. The time needed to develop these assays and their reagents is one of the major bottlenecks for SARS-CoV-2 trials. However, we also cannot sacrifice quality, and assays must be properly designed and validated for their intended use because of the immense public health implications they carry. This article provides a snapshot of the assays needed for SARS-CoV-2 with suggestions for accelerating development and implementation of these assays within analytical and logistical challenges.

At the same time, COVID-19 will likely forever change the field of drug development and bioanalysis. Looking forward, we propose ideas to prepare for a new future where assays must regularly be developed rapidly, and on a global scale.

## Fit-for-purpose SARS-CoV-2 assays for their context of use

Assays and biomarkers are needed for COVID-19 trials for the purposes of patient stratification and enrollment, determining pharmacokinetics (PK), characterizing mechanism of action and measuring therapeutic effect. Human sample assays to detect biomarkers, antibodies and therapeutic responses in patients and *in vitro* assays showing neutralization of the virus are needed. Importantly, the components of the assay (e.g., capture antibodies) and validation of the assay must be tailored to the use of the assay. For each of the assays described below, their intended use determines whether the assay should be quantitative versus quantitative or which components of the virus-host response need to be measured.

## Viral detection/viral load assays

The presence of active SARS-CoV-2 infection must be assessed for enrolling patients who are positive for the virus in trials and assess prevention or improvement in the infection by measuring viral load (viral titer). Similar to diagnostic tests, quantitative polymerase chain reaction (qPCR) should be used to detect SARS-CoV-2 RNA. The virus nucleocapsid primers (N1 and N2), noninfectious positive control and human specimen RNA extraction control available from the Centers for Disease Control and third-party vendors, are crucial components of this assay. The US FDA, based on recent evidence, also believes a validated single viral target SARS-CoV-2 assay could provide an acceptable performance.

The design of the assay will depend on the context of use of the assay. RNA measurement for patient enrollment and screening of clinical staff can be qualitative, while assays trying to show reduction in viral load with therapy should be semiquantitative with a synthetic standard of viral genes containing a known quantity of viral RNA copies.

Key considerations for viral load measurement of SARS-CoV-2 include proper collection, transport, storage and extraction of RNA. For swab methods, a nasopharyngeal collection is preferred over throat swabs because higher viral loads are seen sooner after symptom onset in the nose than in the throat [2]. Other possible noninvasive specimens include saliva, which appears to have a similar viral load to nasal swabs [3] and sputum [4]. SARS-CoV-2 RNA appears in serum only when patients are severely sick [5].

Regardless of the specimen chosen, samples should be placed in recommended transport mediums and stored as recommended (typically up to 72 h at 2–8°C). Options for mediums to store samples include purchased or in-house prepared viral transport mediums and phosphate-buffered saline. Due to the need of rapid results, extraction of viral RNA should be performed with automated methods.

Special considerations should also be given to the accuracy of testing assays and the false negative rate. Suggestions to decrease false negative rate include rigorously standardizing sampling and transport procedures, and the use of TRIzol™ (ThermoFisher, CA, USA) to stabilize the RNA while inactivating the virus. Experts also recommend combinatorial testing with different or repeated viral load assays, different anatomic site sampling such as sputum or bronchoalveolar lavage fluid (BALF) and serology testing for SARS-CoV-2 antibodies [6].

We should also prepare to employ the rapid deployment of qPCR testing currently being used, for future infectious diseases or the evolution of SARS-CoV-2 over the next few months. This requires readiness of properly designed primers and the availability of reagents described above. Bioanalytical scientists should be familiar with *in silico* and *in vitro* tests to demonstrate analytical specificity and exclusivity for molecular experiments. Software such as basic local alignment search tool queries are necessary to generate primers without false positives.

## SARS-CoV-2 antibody assays

Detection of antibodies against SARS-CoV-2 with immunoassays is used qualitatively to determine active or past infection (immunized) necessary for patient selection and quantified for determining if a therapy or vaccine produces antibodies against the virus. Antibody assays must not be used alone for diagnosis and patient enrollment.

Capture ligand design for the immunoassay depends on intended use. Basing the capture antibody on the entire S-spike protein will increase sensitivity of the assay but decrease specificity due to homology with other coronaviruses. On the other hand, using a peptide sequence specific to SARS-CoV-2 will likely miss too many positive antibodies. Using the receptor binding domain (RBD) of the S-spike protein that binds to human ACE2, is likely the best balance [7]. An assay screening serum for convalescent plasma therapy should also use the RBD region as the capture antibody antigen, as antibodies targeting this region are more likely to have virus neutralizing potential. Assays can also be designed against the nucleocapsid, but this is typically only supportive data for a trial and not compulsory.

The type of antibodies detected will depend on the time course of infection and can be selected with different secondary antibodies. IgM and IgG antibodies can be detected approximately 4 days after SARS-CoV-2 infection as a marker of active infection followed by IgA antibodies [8].

The positive antibody controls required to validate antibody assays should use human serum. Controls for system suitability, and day-to-day monitoring can use animal antibodies produced by immunizing against recombinant full-length S-spike protein. In assay validation to determine sensitivity and specificity, human serum from at least 30 patients with past infection should be used. We recommend the use of serum taken before December 2019, if possible, as negative samples.

Generation of antibody reagents in animals for serology assays usually takes 4–9 months. Recombinant antibody library generation can produce scalable antibodies in *E. coli* or cell lines in approximately 2 months. Batch-to-batch consistency and antibody sequencing prevents the need to revalidate assays – a common occurrence when using animal antibodies. Antibodies can be customized with human Fc regions so a single detection antibody can be used for both human serum and animal antibody controls. Making these technologies widespread will make us better prepared for the next pandemic.

## Neutralizing antibody assays

For both vaccines and therapeutics, the antibodies produced are tested for their functional efficacy to neutralize the target virus (e.g., prevents binding of RBD to ACE2). Modern neutralizing assays employ a two-part method with: ligand-binding assays using human serum; and *in vitro* cell-based assays to shorten the time and increase throughput needed for these assays.

For SARS-CoV-2, ligand-binding competitive ELISA methods identify ‘positive’ samples that prevent RBD and ACE2 binding with increasing serum concentrations. Cell-based assays with infectious viral particles are then used to determine if positive serum neutralizes virus entry and replication. Separating the ligand binding and cell-based steps is logistically beneficial as the functional neutralizing assay requires a biosafety level three laboratory, while a screening ligand-binding assay does not.

## Vaccine antigen & antibody assays

For vaccine trials, the viral component antigen in the vaccine must be measured after dosing as a measurement of PK. Current components for vaccines under development include whole live attenuated virus, protein subunits of S-spike protein or the RBD and DNA/RNA vaccines [9]. The assay design and validation must be customized for each vaccine as the antibodies or primers used in the assay must match the vaccine immunogen. A PK assay for a vaccine using only a portion of the S-spike protein should use antibodies against the exact peptide sequence. Assays for vaccines with multiple components or adjuvants should be measured with either a multiplex assay or separate single assays.

Primary potency measurements for vaccine trials include antibody titers against vaccine antigens and determination of antiviral neutralizing activity. These assays can employ strategies for anti-SARS-CoV-2 antibodies and neutralizing antibodies described above. Using the vaccine component as the capture ligand enables detection of relevant antibodies induced by vaccine components.

## Cytokine biomarkers

The release of cytokine biomarkers after presentation of SARS-CoV-2 viral particles on antigen presenting cells initiates a cytokine storm likely responsible for the respiratory complications of the disease [10]. Studies show a hyperinflammatory cytokine storm, with alterations in serum IL-2, IL-6, IL-7, granulocyte-colony stimulating factor, IP-10, MCP-1, MIP1-α and TNF-α, is positively correlated with COVID-19 disease severity and fatality [10].

In SARS-CoV-2 trials, cytokine biomarkers can be monitored for patient enrollment, showing mechanism of action (particularly for anti-inflammatory therapies) and monitoring treatment effect in contexts of use. In previous viral challenge trials with neutralizing antibody therapies, only IP-10 and IFN-g showed significant changes after drug dosing [11]. The specific cytokines needed for SARS-COV-2 trials is yet to be characterized with different studies showing different cytokine profiles. Therefore, larger multiplex panels are recommended, especially those that are well characterized for reliability and speed. Past studies have seen larger changes in cytokines in respiratory-specific matrices such as bronchoalveolar lavage fluid than in serum [12], however assessment in serum is likely most appropriate due to the urgency of the trials.

## Furin cleavage assay

Several other assays can be used as biomarkers to support the mechanism of action of therapies and vaccines. Many viruses use human endogenous proteases/convertases (e.g., furin) to cleave the surface glycoproteins for entry into a cell. The SARS-CoV-2 strain, uniquely uses furin expressed highly in the lung to cleave S-spike protein into functional S1 and S2, which binds to ACE2 [13]. Intracellular furin near the Golgi apparatus is also used to package new viral particles. Vaccines or therapeutic antibodies may block the interaction of S protein with furin or target furin itself. Measurement of furin cleavage activity of S protein can be used for this class of therapeutics as a proof of concept/mechanism of action [14]. This *in vitro* assay with recombinant furin would show a decrease in furin cleavage of S protein after development of neutralizing antibodies that block furin cleavage.

## ELISpot cell-mediated immunity

The antibody responses measured in the assays above characterize B-cell humoral response to infection and vaccination. Cell-mediated immunity should also be characterized for drug development as T-cell release of cytokines after an infection or vaccination promote B-cell maturity. T-cell responses to past coronaviruses have been assessed with enzyme-linked immune absorbent spot (ELISpot) assays [15]. ELISpot functionally assesses the impact of a vaccine on T-cell cytokine secretion. It can cost-effectively screen responses to an entire pathogen proteome and estimate memory response in vaccine recipients.

## Obtaining quality reagents for SARS-CoV-2 assays

Assays for COVID-19 must be developed quickly and scaled to laboratories worldwide, while maintaining rigorous quality because of the implications of the test results. Scientists must therefore ensure reagents such as antibodies are specific for SARS-CoV-2 and be able to source enough quantities needed for the high demand.

Determining whether assays are detecting antibodies against SARS-CoV-2 and not other coronaviruses is crucial, because studies show there is limited cross-reactivity between antibodies for SARS-CoV and SARS-CoV-2 even though they share the same ACE2 binding domain [16]. We should be wary of antibody tests claiming to be reviewed by the FDA, but actually detect past coronavirus infections, because of recently relaxed FDA rules allowing tests to be sold without data review. Using the strategies for developing capture ligands for immunoassays described above can alleviate these concerns.

## Regulatory considerations on an accelerated timeline

With the urgent need for therapeutics, laboratories characterizing SARS-CoV-2 therapies should understand our responsibility in developing assays with wide implications for individual patients and the public. We encourage following guidelines from worldwide regulatory considerations such as public health authorities, existing FDA Bioanalytical Method Validation guidelines, FDA guidelines for clinical trials during the COVID-19 outbreak and having discussions with regulators when needed [17].

There is also ongoing discussion of whether assays to measure biomarkers for drug development should be performed in a Clinical Laboratory Improvement Amendments lab or a Good Laboratory Practice (GLP) lab. Current guidance formed at the 2019 Workshop for Recent Issues in Bioanalysis indicate biomarkers must be tested under CLIA regulations when intended for individual patient treatment (including trial enrollment), but the approach should be reviewed with regulatory agencies [18]. Biomarkers for internal decision making (including trial end points) should follow GLP guidelines.

## Future perspective: how can bioanalytical scientists prepare for a new normal?

At a time when the world is looking to scientists to alleviate the COVID-19 pandemic, bioanalytical scientists can play a pivotal role in developing assays rapidly to bring these therapies to patients faster. The combination of human sample (e.g., anti-CoV-2 antibodies) and *in vitro* assays (e.g., neutralizing antibodies and furin cleavage) presented above may streamline the time and cost of bioanalytical testing to support therapeutic development. The urgent worldwide need for therapeutics will require a sustained capacity of many laboratories to perform these assays.

Beyond COVID-19, we as a community must adapt for a future where drugs must be developed rapidly. It is a matter of when, not if, another pandemic occurs requiring rapid assay development. This requires adopting more biomarkers and *in vitro* assays such as those suggested in this article into trial designs. We should also embrace novel technologies such as recombinant antibodies and combinatorial antibody libraries to reduce lead time for antibody generation.

Finally, we should develop novel surrogate end points for clinical trials, especially vaccine trials that currently can take years to show an end point of population immunity. We can learn from recent history when the incorporation of CD4/CD8 cell ratios and HIV viral load as surrogate end points for HIV successfully accelerated antiviral therapy approval [19]. Validating biomarkers and clinical end points will require continued collaboration between academia, physicians, industry and regulatory agencies. While the current pandemic carries immense responsibility and challenges, the steps we take now will improve drug development for future pandemics and all diseases.
